# Lipid–Polymer Hybrid Nanoparticles for mRNA Delivery to Dendritic Cells: Impact of Lipid Composition on Performance in Different Media

**DOI:** 10.3390/pharmaceutics14122675

**Published:** 2022-12-01

**Authors:** Lena Kliesch, Simon Delandre, Aljoscha Gabelmann, Marcus Koch, Kai Schulze, Carlos A. Guzmán, Brigitta Loretz, Claus-Michael Lehr

**Affiliations:** 1 Helmholtz-Institute for Pharmaceutical Research Saarland (HIPS), Helmholtz Centre for Infection Research, Campus E8.1, 66123 Saarbrücken, Germany; 2 Department of Pharmacy, Saarland University, 66123 Saarbrücken, Germany; 3 Department of Vaccinology and Applied Microbiology, Helmholtz Centre for Infection Research, Inhoffenstraße 7, 38124 Braunschweig, Germany; 4 INM-Leibniz-Institut für Neue Materialien, Campus D2 2, 66123 Saarbrücken, Germany

**Keywords:** mRNA delivery, PLGA, DOTMA, DOPE, hybrid nanoparticle, dendritic cells

## Abstract

To combine the excellent transfection properties of lipids with the high stability of polymeric nanoparticles, we designed a hybrid system with a polymeric core surrounded by a shell of different lipids. The aim is to use this technology for skin vaccination purposes where the transfection of dendritic cells is crucial. Based on a carrier made of PLGA and the positively charged lipid DOTMA, we prepared a panel of nanocarriers with increasing amounts of the zwitterionic phospholipid DOPE in the lipid layer to improve their cell tolerability. We selected a nomenclature accordingly with numbers in brackets to represent the used mol% of DOPE and DOTMA in the lipid layer, respectively. We loaded mRNA onto the surface and assessed the mRNA binding efficacy and the degree of protection against RNases. We investigated the influence of the lipid composition on the toxicity, uptake and transfection in the dendritic cell line DC 2.4 challenging the formulations with different medium supplements like fetal calf serum (FCS) and salts. After selecting the most promising candidate, we performed an immune stimulation assay with primary mouse derived dendritic cells. The experiments showed that all tested lipid–polymer nanoparticles (LPNs) have comparable hydrodynamic parameters with sizes between 200 and 250 nm and are able to bind mRNA electrostatically due to their positive zetapotential (20–40 mV for most formulations). The more of DOPE we add, the more free mRNA we find and the better the cellular uptake reaching approx. 100% for LPN(60/40)–LPN(90/10). This applies for all tested formulations leading to LPN(70/30) with the best performance, in terms of 67% of live cells with protein expression. In that case, the supplements of the medium did not influence the transfection efficacy (56% vs. 67% (suppl. medium) for live cells and 63% vs. 71% in total population). We finally confirmed this finding using mouse derived primary immune cells. We can conclude that a certain amount of DOTMA in the lipid coating of the polymer core is essential for complexation of the mRNA, but the zwitterionic phospholipid DOPE is also important for the particles’ performance in supplemented media.

## 1. Introduction

The mRNA-based vaccines have been put in the spotlight since the COVID-19 pandemic which consequently led to tremendous progresses in knowledge about beneficial mRNA-modifications in combination with lipid-based carrier systems. One possible next development step to further advance the immune response might be the change of the vaccine application site from the deltoid muscle to more immune rich tissues like mucosae or skin. As those epithelia represent the outermost biological barrier of the human body, defense mechanisms against potentially harmful components are in place. This includes for example a plethora of resident immune cells and secreted defense molecules like RNases that degrade viral RNA molecules [1,2]. Such an environment is on the one side a chance for stronger immune responses, but at the same time it challenges a potential vaccine candidate’s stability and thereby requires a sufficient degree of protection of the nucleic acid cargo against degradation enzymes [3,4].

Lipid–polymer nanoparticles (LPN) have the potential to fulfill these demands as they combine the high stability of polymeric nanoparticles with the good nucleotide delivery and transfection properties of lipids. Su et al. showed that hybrid nanoparticles made of poly(β-amino ester) (PBAE) and phospholipids bind and thereby protect the mRNA electrostatically bound to the surface [5]. Their nanocarriers transfected around 30% in vitro and showed significant protein levels after intranasal application in vivo demonstrating the added value of such nanoparticle formulations. The systematic comparison of mRNA loaded nanoparticles either made of a cationic biopolymer (protamine) or lipids (DOTAP) with combined nanocarriers (mixture of protamine and DOTAP) revealed better transfection rates for the lipid–polymer nanoparticles [6]. In our lab, Yasar et al. tested cationic polymer-polymer nanoparticles against lipid–polymer nanoparticles, both based on a poly(lactic-*co*-glycolic acid) (PLGA) core and confirmed again the beneficial effects of such hybrid nanoparticles.

To further optimize that lipid–polymer-system and to cope with the potential toxic effects of the used cationic lipid 1,2-di-*O*-octadecenyl-3-trimethylammonium propane (DOTMA) [7], we decided to vary the composition of the lipid layer and to systematically investigate its effect. Keeping the cationic lipid for the complexation of mRNA, we added different amounts of the phospholipid 1,2-dioleoyl-*sn*-glycero-3-phosphoethanolamine (DOPE) to the lipid layer as this phospholipid is well known for its helper lipid activities and the destabilization of the endosome membrane favoring endosomal escape [8].

In this study, we aimed to identify the optimum LPN composition regarding colloidal stability, mRNA protection, cytocompatibility and transfection efficiency. Therefore, we prepared nanoparticles with different ratios of DOTMA and DOPE in the lipid layer surrounding the PLGA core and exposed them to RNase A for studying the degradation kinetics. We then evaluated the performance in protein and salt containing media to select the best candidate with the most stable results under various conditions. Finally, we challenged that system with primary mouse dendritic cells and checked their ability to process and present the antigen, thereby leading to the activation of antigen-specific T-cells.

## 2. Materials and Methods

### 2.1. Preparation of Lipid–Polymer Nanoparticles and mRNA Loading

**Figure 1:**
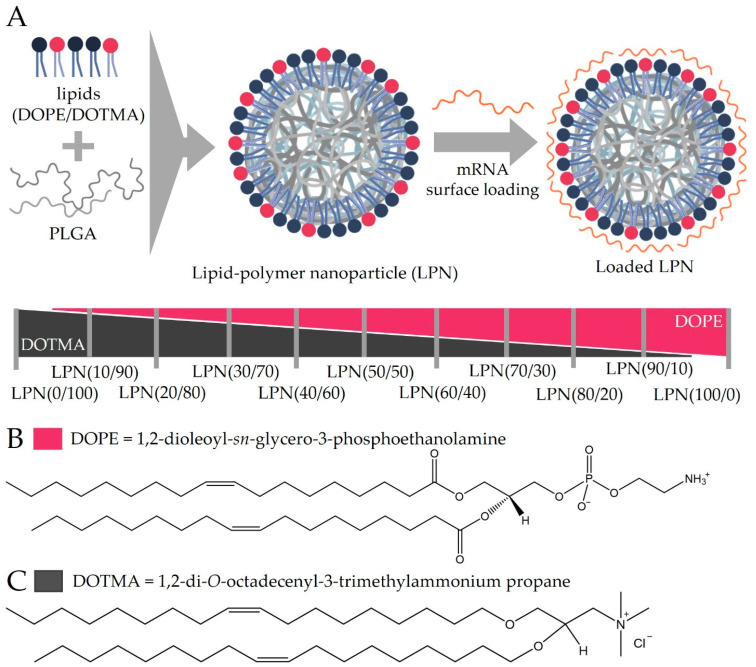
(**A**): Anticipated nanocarrier structure. The values in brackets represent the molar percentage of each lipid that is present in the lipid layer. The first number refers to the DOPE, the second to the DOTMA content. (**B**): structure of DOPE (1,2-dioleoyl-*sn*-glycero-3-phosphoethanolamine), (**C**): structure of DOTMA (1,2-di-*O*-octadecenyl-3-trimethylammonium propane).

All used nanoparticles were produced with the same method based on a previously published protocol [9]. Each particle formulation contains a PLGA core (Resomer RG 503H, 50:50, Evonik Industries Ag, Darmstadt, Germany) and a lipid layer consisting of DOTMA and/or DOPE in different ratios. Both lipids were purchased from Avanti polar lipids (Birmingham, AL, USA). Sigma-Aldrich (Darmstadt, Germany) supplied the chloroform to dissolve all compounds. Figure 1 shows a schematic of the LPN components and explains the nomenclature.

For preparing a new particle batch, DOPE was dissolved in chloroform (25 mg/mL), DOTMA was purchased as chloroform solution (25 mg/mL). Aliquots containing up to 3.5 mg DOPE were mixed with different molar amounts of DOTMA keeping the overall number of nitrogen atoms constant to enable a consistent number of potential mRNA binding sites. Chloroform diluted the lipid mixture up to a total volume of 250 µL. PLGA was dissolved in chloroform as well (30 mg/mL). An aliquot of 250 µL was then added to the lipids and mixed thoroughly by vortexing. After adding 250 µL of ultra-purified water (Milli-Q^®^, Merck Millipore, Darmstadt, Germany) (MQ) to the organic phase, the emulsion was mixed by ultrasound sonication (Branson Ultrasonic Corporation, Brookfield, CT, USA) for 30 s at an amplitude of 30%. Without any break, 1 mL of polyvinyl alcohol (PVA) in MQ (2% *w*/*v*, Mowiol 4-88, Sigma-Aldrich, Darmstadt, Germany) was introduced and homogenized by another 30 s sonication. The resulting emulsion was poured into 5 mL PVA-solution and stirred for 3 h to evaporate the chloroform. LPNs were stored in the fridge until usage.

To prepare fluorescently labeled nanoparticles, DiD (1,1-dioctadecyl-3,3,3,3-tetramethylindodicarbocyanine, 10 mg/mL in ethanol, life technologies, Invitrogen) or fluoresceinamine was used. In case of DiD, 9 µL of the dye were added to the PLGA before mixing with the lipids resulting in a non-covalent incorporation into the nanoparticle. Unbound dye was washed away using a 100 kDa dialysis membrane for 24 h under light protection. For fluorescein labeling, PLGA was covalently coupled to fluoresceinamine (Sigma-Aldrich, Darmstadt, Germany) following the published protocol [10]. This modified PLGA substituted the unlabeled polymer during the preparation.

### 2.2. Measurement of Particle Properties

Physicochemical properties such as hydrodynamic size, polydispersity index (PDI) and zetapotential of nanoparticles were investigated using dynamic light scattering (DLS; Zetasizer Nano, Malvern Instruments, Malvern, UK). Before measurement, an aliquot of LPNs was diluted 10-fold in deionized water and measured three times resulting in mean values and standard deviations for each batch.

For the assessment of the stability in supplemented medium, DiD-labeled LPNs were loaded with mRNA and then incubated in the same concentrations as we used for transfection experiments at 37 °C. As comparison to the medium, deionized water was used. At the beginning, after 4 and 24 h an aliquot was taken and diluted 5-fold in water to stop any ongoing processes. Samples were recorded in three videos of 30 s each using the fluorescence channel of the nanoparticle tracking analysis (NanoSight LM10, Malvern Instruments, Worcestershire, UK). The video tracks were analyzed using the NanoSight 3.3 software.

### 2.3. Cryo-TEM

For cryo-TEM, a 3 µL droplet of the LPNs dispersion was placed on a holey carbon supporting TEM grid (Plano, Wetzlar, Germany, type S147-4), blotted for 2 s and plunge-freezed in undercooled liquid ethane using a Gatan (Pleasanton, CA, USA) CP3 plunge freezer operating at −165 °C. The vitreous sample was transferred under liquid nitrogen to a Gatan model 914 cryo-TEM sample holder and investigated by TEM (JEOL, Akishima, Tokio, Japan, model JEM-2100 LaB_6_) bright field imaging at 200 kV accelerating voltage under low-dose conditions. Imaging was performed using a Gatan Orius SC1000 CCD camera and an exposure time of 4 s.

### 2.4. mRNA Binding and Release

If not stated otherwise, mRNA encoding for the fluorescent reporter protein mCherry was used (CleanCap™ mCherry mRNA (5moU); TriLink BioTechnologies LLC, San Diego, CA, USA) with a stock concentration of 1 µg/µL. Following the findings of Yasar et al. for DOTMA-PLGA-LPN(LPN(0/100)), a weight ratio of 1:20 was selected which corresponds to a N:P ratio of 2.81. This ratio between all nitrogen atoms (N) of the lipids and the phosphates (P) in the mRNA backbone is the same for all formulations to enable good comparison. Calculated weight ratios differ among particle types due to different molecular weights of the lipids resulting in different particle stock concentrations.

The actual loading of mRNA onto the positively charged surfaces of the nanocarriers was performed by carefully mixing the particle suspension with the appropriate amount of mRNA with a pipette and incubation for one hour at room temperature (RT).

To confirm the successful complexation of mRNA, a gel retardation assay was performed using a 0.7% *w*/*v* agarose gel (molecular biology grade, Serva, Heidelberg, Germany) and a 1× Tris-Borate-EDTA (TBE) buffer. After mixing samples with orange loading dye (Thermo Fisher Scientific, Waltham, MA, USA), samples containing 400 ng mRNA were loaded into the gel. After gel electrophoresis at 60 V for 30 min, ethidium bromide (35 microgram per gel, stock 10 mg/mL in water, Sigma-Aldrich, Darmstadt, Germany) enabled the detection of the nucleic acids with the Fusion FX7 UV illuminator (Peqlab, Erlangen, Germany).

Aiming for the detachment of the mRNA from the nanoparticles’ surfaces, High Molecular Weight heparin (H3393-100KU, Grade I-A, low-molecular weight Sigma-Aldrich, Darmstadt, Germany) was dissolved in MQ (30 mg/mL in MQ). To competitively occupy mRNA binding sites and thus release the nucleic acids, 375 µg of dissolved heparin was added per microgram mRNA to the nanoparticles for 30 min at RT.

Complementing the gel electrophoresis, we used the Quant-iT™ RiboGreen^®^ RNA quantification kit (Molecular Probes, Inc., Eugene, OR, USA) according to the manufacturer’s protocol. In brief, LPNs freshly loaded with mRNA were diluted 1:100 in Tris-EDTA (TE)- buffer and mixed with RiboGreen^®^ dye (1:200 in TE-buffer) in equal amounts up to 200 µL in a black 96-well plate. After 5 min incubation, fluorescence was measured with a plate reader (excitation 480 nm, emission 520 nm; Infinite 200 Pro, Tecan Austria GmbH, Grödig, Austria). The binding efficacy was calculated relative to the fluorescence of naked mRNA measured in the same experiment.

### 2.5. RNase Exposure and mRNA Protection

The degree of protection of the loaded mRNA against nucleases is a very important feature for nanocarriers. Therefore, we investigated how much mRNA is degraded after RNase A (DNase and Protease free, EN0531, Life Technologies Inc., Carlsbad, CA, USA) addition over the course of 30 min. A setup like the previous mRNA quantification with RiboGreen^®^ was used but this time diluted LPN-samples were mixed with a higher concentration of the RiboGreen^®^ dye (1:100 in TE-buffer). To avoid any delays during the initialization process of the kinetic experiment and to enable short measurement intervals, we used the automatic dispensing function of our plate reader to add the RNase A (in TE-buffer) to the samples in the 96-well plate. The final RNase concentration was 0.0013 Kunitz (K)/µg mRNA. Immediately after the enzyme dispensation, the device automatically started measuring the fluorescence of the samples once a minute over a course of 30 min. Then, RiboLock^®^ RNase Inhibitor (10 U/ng RNase protein, Thermo Fischer Scientific, VilRiboLock^®^ weight, 50 mg/mL in MQ) released the remnant mRNA from the nanoparticles. For both steps, we continued measuring every minute for 30 min in the plate reader. Measured values for the free mRNA were corrected with TE-buffer blanks. For the LPNs, we used plain, unloaded nanoparticles of each kind undergoing the same treatment as the mRNA loaded ones as blanks to reduce unspecific dye binding effects as much as possible. These calculated values were compared between samples treated either with RNase A or with TE-buffer instead of the digestion enzyme, followed by RiboLock^®^ and heparin additions for all samples.

In parallel, aliquots of the loaded LPN samples were treated with the same concentrations of enzymes without measuring fluorescence. At the end, these samples were transferred to a gel containing RiboGreen^®^ (1:2000 in agarose (0.7% *w*/*v*)). The gel was run at 60 V for 60 min and analyzed with a fluorescence lamp (Fusion FX7, Peqlab, Erlangen, Germany).

### 2.6. Cell Culture

To evaluate the performance of the nanocarriers in vitro, we selected the murine bone-marrow derived dendritic cell line DC2.4 (SSC142, Merck Millipore, Darmstadt, Germany). The cells were maintained in complete medium based on RPMI (Roswell Park Memorial Institute; 1640 1×, Ref 21875, Gibco/Life Technologies, Paisley, UK) supplemented with 10% FCS (fetal calf serum, Ref 10270106, Gibco, Paisley, UK), HEPES buffer (4-(2-hydroxyethyl)-1-piperazineethanesulfonic acid; 0.01M, Gibco, Paisley, UK), non-essential amino acids (1×, Gibco, Paisley, UK) and β-mercaptoethanol (0.0054×) and cultivated in a humidified incubator at 37 °C with 5% CO_2_.

### 2.7. In Vitro Studies with DC2.4

50,000 DC2.4 cells were seeded per well in 500 µL cultivation medium in a 24 well plate and incubated for 48 h at 37 °C and 5% CO_2_. On the day of experiment, FITC-labeled nanoparticles were loaded with mRNA. During the incubation time, the cells were prepared by washing twice with prewarmed HBSS (Hank’s Balanced Salt Solution, Gibco, Paisley, UK) under the laminar flow bench and by adding either plain RPMI (no phenol red, 1640, Ref 11835, Gibco, Paisley, UK) or regular growth medium including all mentioned supplements such as FCS or HEPES. In the next step, the loaded nanocarriers were added to reach a final concentration of 1 microgram mRNA per well which corresponds to a concentration of 40 µg/mL for the LPN(0/100). Cells were incubated with the particles at 37 °C for 24 h under slow shaking. We used ethanol (5% in HBSS) as dead and medium as live control.

The next day, we washed the cells twice with HBSS, detached them with trypsin-EDTA and stopped the process with FACS-buffer (2% FCS in HBSS). After transferring the cell suspensions to round-bottom FACS tubes, we centrifuge the DCs for 5 min at 300× *g* and 4 °C followed by another washing step with PBS. The resulting pellet was resuspended in the staining solution containing a dye to allow viability assessment (DAPI 1:1000 in PBS, stock 1 mg/mL, Sigma-Aldrich, Darmstadt, Germany) and then incubated for 20 min at room temperature under light protection. Samples were directly measured using flow cytometry (BD LSR Fortessa Cell Analyzer, Biosciences, Heidelberg, Germany). 20,000 events were counted per sample and later analyzed with the FlowJo Software (FlowJo 10.8.1, FlowJo, OR, USA). For the analysis of the potential cytotoxic effects of the nanocarriers, we compared the percentages of live cells in the entire measured cell population excluding cell debris and DAPI-positive, dead cells for each formulation. To evaluate the transfection efficacy two parameters were calculated. Firstly, the percentage of cells expressing the mCherry protein in the entire population and secondly, the percentage of transfected cells in the live cell population. In parallel, we investigated how many of the live cells are positive for the green signal coming from the FITC-nanocarriers indicating successful uptake.

### 2.8. Generation of Bone-Marrow-Derived Dendritic Cells (BMDC)

Mice femur and tibial bones from 6–8-week-old C57BL/6 (Envigo, Borchen, Germany) were flushed with culture medium (RPMI 1640 supplemented with 10% fetal calf serum, 100 U/mL penicillin, 50 µg/mL streptomycin, 100 µg/mL gentamycin; Gibco, Paisley, UK) to collect bone marrow cells. Erythrocytes were lysed in ACK lysing buffer (Ammonium-Chloride-Potassium, Sigma-Aldrich, Darmstadt, Germany). Bone marrow cells were then seeded at 1.10^6^ cells/mL in culture medium supplemented with 5 ng/mL murine GM-CSF (BD Biosciences, San Diego, CA, USA) and cultured for 7 days.

### 2.9. In Vitro LPN Transfection and Antigen-Specific T-Cells Activation

For experiments with the generated mouse BMDCs, mRNA encoding for the model antigen ovalbumin (CleanCap™ mCherry OVA (5 moU); TriLink BioTechnologies LLC, San Diego, CA, USA) was used keeping the same N:P ratio as for mCherry mRNA. After 3 h incubation in OptiMEM, transfection medium was changed and replaced by culture medium (RPMI 1640 supplemented with 10% fetal calf serum, 100 U/mL penicillin, 50 µg/mL strep-tomycin, 100 µg/mL gentamycin; Gibco, Paisley, UK) for further 20 h. After 24 h, OVA-specific CD8^+^ T-cells isolated from spleen and lymph nodes of OT-I mice (OVA-TCR transgenic mice OT-I (C57BL/6-Tg(TcraTcrb)1100Mjb/J) bred at the animal facilities of Helmholtz Centre for Infection Research under specific pathogen-free conditions) were labeled with 10 μM carboxy fluorescein succinimidyl ester (CFSE; Molecular Probes, Eugene, MO, USA) and co-cultured for 6 days with the LPN-stimulated murine BMDCs. Afterwards, cells were collected and the loss of CFSE signal in CD8^+^ OT-I cells in response to OVA peptide presentation by BMDC was determined by flow cytometry using a BD Fortessa and analyzed with FlowJo software v10.5.

### 2.10. Statistical Analysis

Statistics were calculated with the GraphPad Prism 9 software, using two-way ANOVA followed by a Tukey’s multiple comparison test. A *p* value below 0.05 was considered significant (* *p* < 0.05; ** *p*< 0.01; *** *p* < 0.001; **** *p* < 0.0001). N represents the number of biological replicates in independent experiments and n the number of technical replicates for all the experiment.

## 3. Results

### 3.1. Preparation and Characterization of Nanocarriers

**Figure 2:**
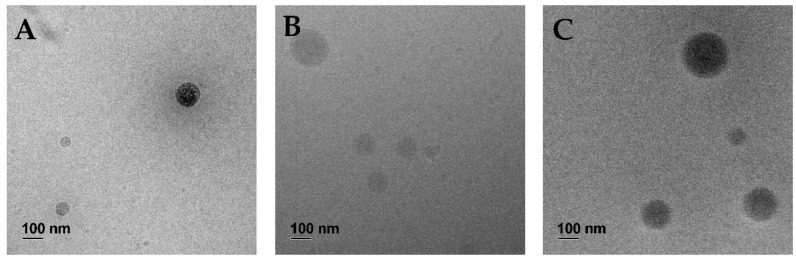
Cryo-TEM images of plain lipid–polymer nanoparticles: (**A**) DOTMA-PLGA-LPN (LPN(0/100)), (**B**) LPN(50/50), (**C**) DOPE-PLGA-LPN (LPN(100/0)). Images confirm spherical shape.

**Figure 3:**
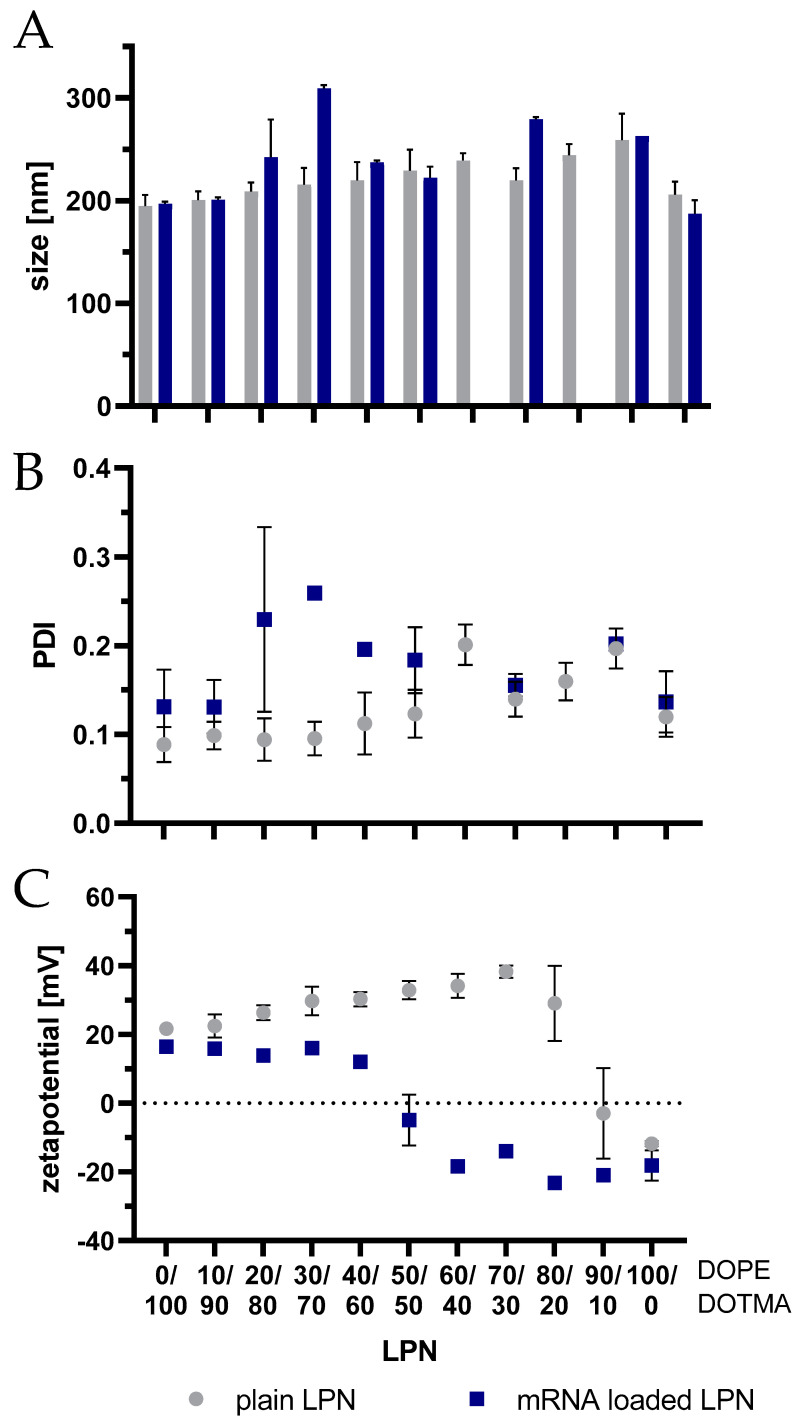
Physicochemical parameters of lipid-polymer nanoparticles (LPN) with and without complexed mRNA. (**A**) size in nm, (**B**) polydispersity index (PDI), (**C**) zetapotential in mV. Data is displayed as mean and standard deviation between batches (3–5 batches for plain, 1–2 batches for loaded LPNs). Plain LPNs have comparable sizes and PDI. The more DOPE the nanoparticles contain, the higher is the measured zetapotential of the plain LPN. Inversion of zetapotential after surface loading of mRNA onto LPNs with at least 50% DOPE in lipid layer.

We used the same protocol to produce all presented lipid–polymer nanoparticles (Figure 1A). While keeping the PLGA amount and added volumes constant, we varied the molar ratio of the lipids DOPE (Figure 1B, indicated by the first number within the brackets) and DOTMA (Figure 1C, second number). The selected ratios ranged from DOTMA-only LPN (LPN(0/100)) to LPNs with only DOPE in the lipid layer (LPN(100/0)). All tested nanocarriers are displayed together with the used nomenclature and a schematic drawing in Figure 1. Independent of the composition, this batch-based preparation method resulted in reproducible and homogenous nanocarriers with a round shape as confirmed by cryoTEM imaging (for representative images see Figure 2). The hydrodynamic sizes ranged from 195–260 nm with a PDI below 0.2 (Figure 3A,B). The preparation process was very robust, as demonstrated by the minimal inter-batch differences shown by the standard deviations. Additionally, an overlay of single measurements for each batch of LPN(70/30) is shown in Figure S1. The zetapotential of the plain LPNs raised with the increased DOPE content, reaching its maximum value for the LPN(70/30) and eventually dropped, leading to a negative value of around −12 mV for the DOPE-only LPN(LPN(100/0) (Figure 3C).

### 3.2. Assessment of mRNA Loading

The N:P ratio of 2.81 was constant for all nanocarriers and resulted in loaded LPNs with a slightly higher PDI compared to the plain nanoparticles and similar sizes in most cases. For LPN(20/80) and LPN(30/70) the mean size increased to 240 nm and 310 nm, respectively with a PDI above 0.2 (Figure 3A,B). LPN(70/30) showed a mean size of 280 nm while maintaining the low PDI of 0.14. The measurement of the surface charge revealed the expected reduction of the positive charge after loading of the negatively charged mRNA onto the nanoparticles’ surfaces. The more DOPE the LPN contained, the larger was the difference between plain and mRNA loaded LPNs with a maximum value of 52 mV for LPNs (60/40–80/20), respectively. While the LPNs (0/100–40/60) showed a reduced but still positive zetapotential in the loaded state, LPNs with a DOPE content of at least 50% in the lipid layer converted to a negative surface (−5 mV for LPN(50/50)) when loaded. For LPNs (60/40–90/10) we measured negative values between −14 mV down to −23 mV. The LPN(100/0) kept the negative surface potential indicating limitations for mRNA loading.

**Figure 4:**
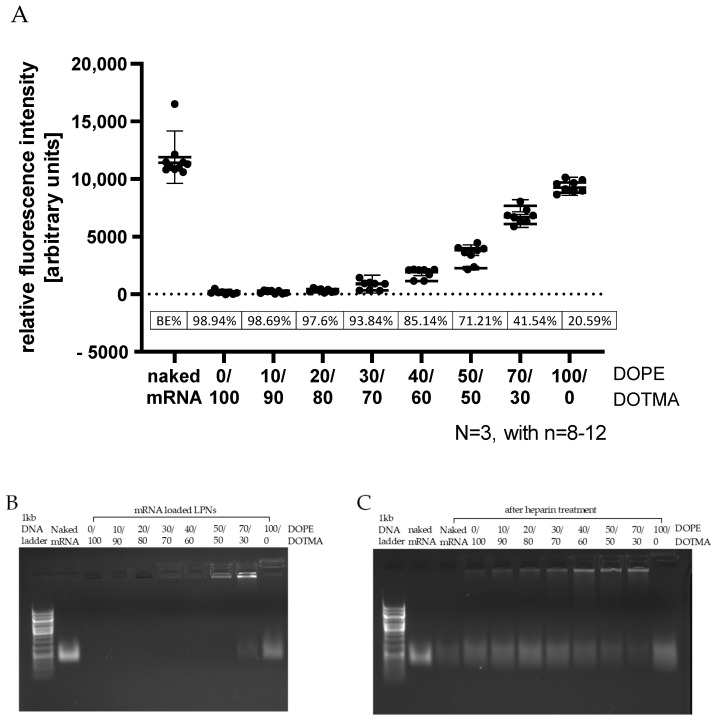
mRNA binding efficacy of LPN. (**A**): Quantification of mRNA fraction accessible for RiboGreen^®^ dye binding and binding efficacy relative to naked mRNA. Increasing the percentage of DOPE (>20%) decreases the packing density of mRNA on LPNs. (**B**): Gel electrophoresis of mRNA-loaded LPNs. Effect of increased DOPE amount to reduce binding strength is confirmed. (**C**): Gel electrophoresis of mRNA-loaded LPNs following heparin treatment. Polyanion competition releases mRNA partially and is dependent on the lipid ratio.

To further confirm successful loading of the mRNA onto the surfaces of the nanoparticles, we performed a RiboGreen^®^ assay following the manufacturer’s protocol. After binding the fraction of accessible mRNA, the added dye exhibits a green fluorescence that can be measured with a plate reader. Figure 4A shows the measured fluorescence intensities and the binding efficacy (BE) calculated relative to the fluorescence of naked mRNA. The more DOPE the LPNs contained, the higher the measured signal, the larger the accessible fraction of mRNA and consequently the lower is the calculated binding efficacy. Up to 40% DOPE content (LPN(40/60)) the LPNs have a calculated BE above 85%, for LPN(70/30) the value is only half of it (42%) and finally only a quarter (21%) for the LPN(100/0). The gel electrophoresis showed a similar trend (Figure 4B). For the nanoparticles with higher DOPE content, the fraction of free mRNA increased. This confirms that at least some parts of the accessible fraction in the RiboGreen^®^ experiment is not bound at all and can thus move out of the gel pocket to the same height as the naked mRNA of the control group. In addition to that signal of free mRNA, we observed a second signal in the gel pockets for these LPNs. We hypothesized that these bands indicate that some part of the mRNA is attached to the surface tightly enough to hold it back from invading the gel but at the same time loosely enough to give room for the ethidium bromide to intercalate. Looking at the other end of the row, we saw that most LPNs containing more DOTMA (0/100–40/60) do not have any signal on the gel–neither free mRNA nor bound mRNA in the pockets. This observation can be explained by a stronger interaction of DOTMA lipids and the mRNA leaving no room for the dye intercalation and thus the lack of any detectable signal. To proof that the mRNA is still there and intact, we added heparin 30 min before we started the electrophoresis. The gel image in Figure 4C shows a free mRNA band for all LPNs and a faint signal in all pockets representing a fraction of mRNA that is bound loosely enough to give room for the ethidium bromide but that is still attached to the nanoparticle surface preventing movement out of the pocket into the gel.

### 3.3. Assessment of mRNA Protection

**Figure 5:**
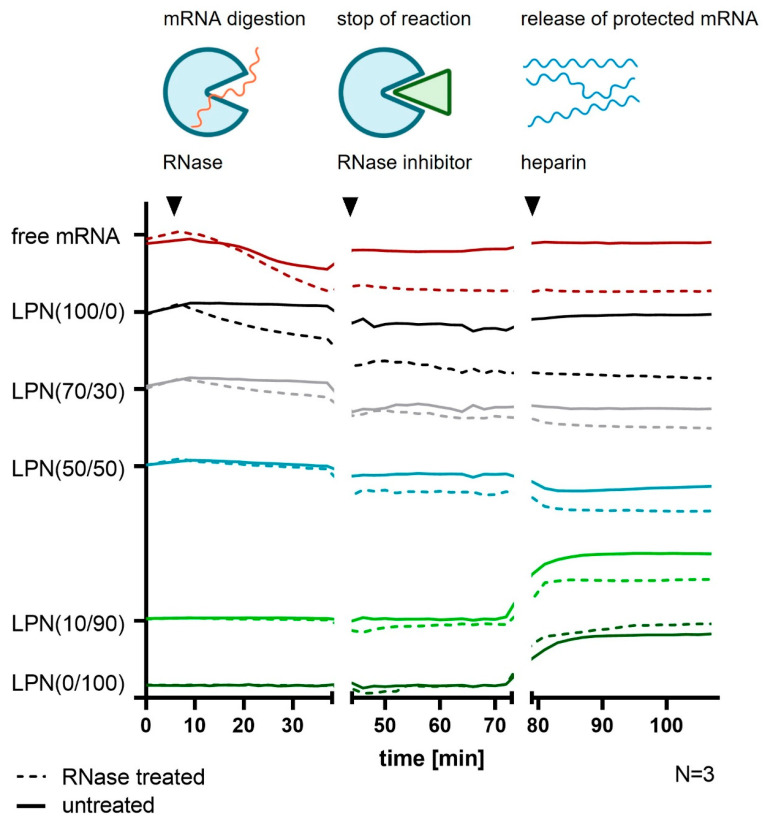
RiboGreen^®^ assay for RNase A degradation. mRNA loaded LPNs were monitored for the fluorescence intensity of RiboGreen^®^ binding to accessible mRNA while sequentially exposed to RNase A and heparin. mRNA protection against RNase A is stronger with higher DOTMA percentage, while the mRNA release upon heparin addition shows less dependency on DOTMA. Solid lines represent untreated samples, dotted lines the RNase treated ones of the same kind. Colors represent the different LPN samples as named. Y-axis shows measured fluorescence intensities of RiboGreen^®^ in arbitrary units for each sample separately to display differences between the two treatment groups. Samples were added to one y-axis to enable comparison of curves. Hight of sample on y-axis is not connected to measured fluorescence values.

The dye-based experiments were able to show that a part of the bound mRNA is accessible for the dyes. However, the more important feature for nanocarriers is their degree of protection of the mRNA molecules against ubiquitous RNases. To quantify that property, we developed an automated plate reader assay based on RiboGreen^®^ where we added RNase A to the samples and followed the mRNA degradation by measuring the intensity loss of fluorescence signal. After stopping the reaction with the specific inhibitor RiboLock, we added heparin to detach the mRNA from the nanoparticles and evaluated the differences of the endpoints between RNase treated and not treated samples. For the LPN(100/0) the difference between the two treatment groups is even higher than for naked mRNA indicating that the mRNA is not at all protected (Figure 5). The LPN(0/100) with only DOTMA in the lipid layer protects the best shown by similar values for both sample groups. For all other tested nanoparticles, the differences are comparable. We can thus conclude that the degree of protection is also in the same range even though LPN(70/30) showed more accessible mRNA in the previous experiments. Heparin increased the fluorescence only in LPN(0/100) and LPN(10/90) and released intact mRNA from the nanoparticles. For other samples, heparin did not show an effect. Thus, the differences at the end of the experiment are mainly due to RNase degradation during the first part of the assay.

### 3.4. In Vitro Experiments with the DC Cell Line

To evaluate the impact of the measured mRNA protection, we incubated the LPNs with the murine dendritic cell line DC2.4. In a first setup, we incubated the cells for 4 h with the plain nanocarriers and saw no hints for cytotoxic effects even with concentrations up to 160 µg/mL (Figure S2). That enabled us to extend the incubation time to 24 h with the LPNs. When using RPMI medium without any supplements and a mRNA concentration of 2 µg/mL enabling a good ratio for transfection between mRNA amount and cells, the comparison of plain LPNs with mRNA loaded ones showed no significant differences (Figure S3). Thus, we decided to test only the loaded LPNs in that concentration in combination with the 24 h time point.

**Figure 6:**
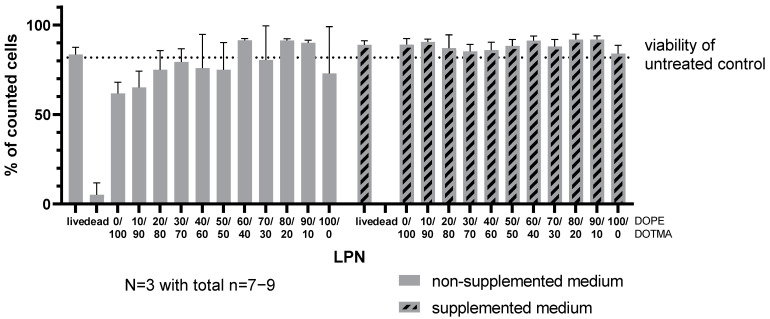
Viability assay. Assessment of cell membrane integrity by DAPI staining of the dendritic cell line DC2.4 after 24 h of incubation with mRNA-loaded LPNs in non-supplemented RPMI-medium vs. optimal growth medium. 5% Ethanol in HBSS (*v*/*v*) was used as dead control, medium without nanoparticles as live control. Analysis of cell population was performed by flow cytometry. This data set was acquired in the same experiment as the uptake and transfection. The mRNA concentration is 2 µg/mL. Only samples with high DOTMA concentration and in non-supplemented medium showed slight cell membrane damage.

**Figure 7:**
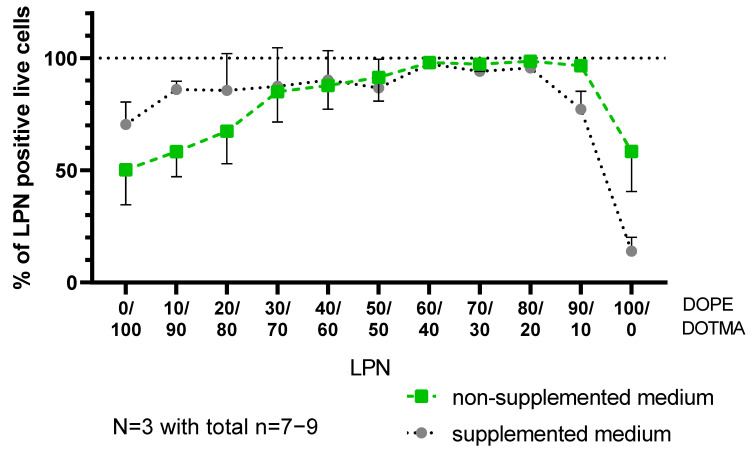
Cellular uptake of LPNs. Percentage of viable DC2.4 cells that show a green fluorescence signal after 24 h incubation with FITC-labeled, mRNA loaded LPN in different media. LPNs with higher DOPE content showed improved uptake with a maximum at LPN(80/20) and (90/10) depending on the medium. DOPE-only LPN have a reduced uptake in both media. Differences between media conditions are non-significant of LPNs (30/70)–(80/20).

To further increase the challenge for the nanocarriers, we selected an incubation in either plain RPMI medium or RPMI supplemented with fetal calf serum, HEPES buffer, β-mercaptoethanol and non-essential amino acids representing the regular growth medium for the DC2.4 cells in our lab. To collect as much information as possible within one experiment, we used FITC-labeled LPNs and loaded them with mRNA encoding for the fluorescent protein mCherry. Table S1 shows that the fluorescent LPNs have sizes, PDIs and zetapotentials similar to the unlabeled ones. To assess the cytotoxicity of the samples and exclude dead cells, we added DAPI as cell viability dye to the single cell suspension during the preparation procedure for flow cytometry. Figure S4 shows the applied gating strategy that resulted in the data points for cytotoxicity, uptake and transfection analysis. The toxicity graph (Figure 6) represents the percentage of live cells in the entire measured cell population excluding cell debris and DAPI-positive dead cells. In parallel, we investigated how many of the live cells are positive for the green signal coming from the FITC-nanocarriers (Figure 7) indicating successful uptake.

In coherence with previously described experiments, the DCs tolerated all LPNs well, especially if the LPNs are dispersed in supplemented medium (Figure 6). Under lower nutrition, less cells survived with LPN(0/100) and LPN(10/90) treatment, but the differences were not statistically significant.

In the next step, we could see a clear trend that the more DOPE the LPN contains the more live cells take up the nanoparticles (Figure 7) in plain medium. The medium supplements increased the uptake for LPNs with lower DOPE content (LPNs 0/100–20/80) but did not show an effect for LPNs (30/70)–(90/10), where uptake was similarly high in both media. LPN(90/10) and LPN(100/0) showed a different trend: while uptake was higher in the non-supplemented medium, we observed reduced and almost no uptake in full medium, respectively. A graph with measured fluorescence intensities indicating the amount of particles associated with the cells is shown in Figure S5.

**Figure 8:**
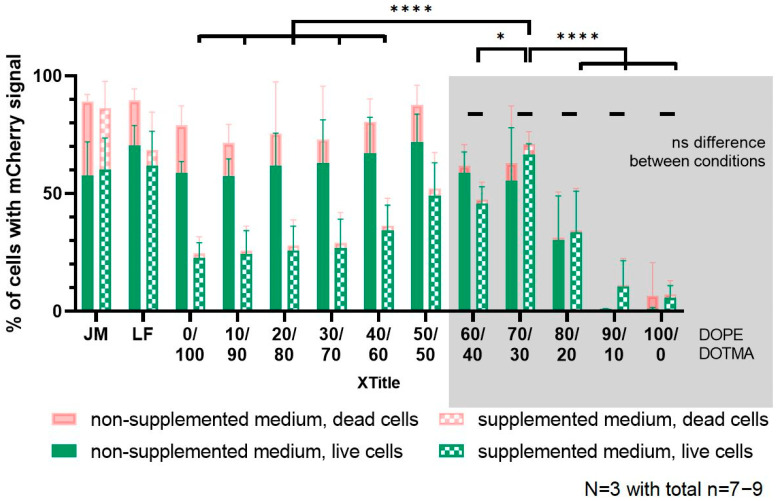
Transfection efficacy in dependence of the medium supplementation. DC2.4 cells were transfected with FITC-labeled LPNs loaded with mCherry mRNA for 24 h in plain RPMI or RPMI supplemented with 10% FCS, HEPES-buffer, non-essential amino acids and β-mercaptoethanol. Each bar shows an overlay of live transfected cells (green bars) and transfected cells in the entire, acquired cell population (pink bars). DAPI staining was used to identify cell membrane damage and to distinguish between live and dead cells. Commercial transfection reagents JetMESSENGER (JM) and Lipofectin (LF) as control samples. Differences between medium conditions get insignificant at samples with DOPE content ≥60% (grey box). Ratio 70/30 is the optimum sample for good transfection efficacy independent of the presence of serum. (* *p* < 0.05; **** *p* < 0.0001).

The third readout of the experiment was the quantification of the cells expressing the mCherry protein in the entire and the live cell population in the two medium conditions. Each bar in Figure 8 shows an overlay of live transfected cells (green bars) and transfected cells in the entire, acquired cell population in red. In non-supplemented medium LPN(0/100) transfected 80% of measured cells but only 60% in the live cell population. The experiment revealed the trend that the more DOPE the LPNs contain, the higher is the percentage of live transfected cells climaxing in 72% for LPN(50/50) until the LPN(60/40) showing lower values again in the plain medium. In the supplemented medium, we observed almost now differences between the total cell population and the live cells even though the percentages of successfully transfected cells were in general lower, especially for LPNs with low DOPE content. Furthermore, the described trend with increasing DOPE amount was extended in the supplemented medium reaching its maximum value of 66% for live transfected cells with LPN(70/30). For LPN(0/100) the transfection of live cells dropped from around 60% before supplementation to 23% afterwards. Beginning with the LPN(60/40), there was no significant difference anymore between the two medium conditions. In comparison with the other nanoparticles in full medium, LPN(70/30) performed significantly better than all other tested LPNs except the LPN(50/50). Furthermore, the transfection of live cells for both LPN(50/50) and LPN(70/30) in the more complex medium was as good as the one of the transfection controls Lipofectin and jetMESSENGER (JM), whereas all other nanoparticles (namely LPN 0/100–40/60, 60/40, 80/20–100/0) deviated significantly. Additionally, the LPN(60/40) showed equal results like Lipofectin for live cells in supplemented medium. The commercial controls showed a stable transfection in both media but with a larger fraction of dead cells. LPN(100/0) did not show transfected cells under any condition. Figure S6 shows the measured fluorescence intensities for the mCherry signal indicating the produced amount and confirming the same trend as the percentage of successfully transfected cells.

After successful transfection in the supplemented medium, we tested if colloidal stability might be the reason for the reduced transfection efficacy. We compared the behavior of LPN(10/90) to LPN(70/30) in MQ and supplemented medium and found no hints for instability (Figure S7) using nanoparticle tracking analysis and DiD-labeled LPNs (check Table S1 for DLS results of DiD-LPNs).

### 3.5. Immune Stimulation in Primary Cells

**Figure 9:**
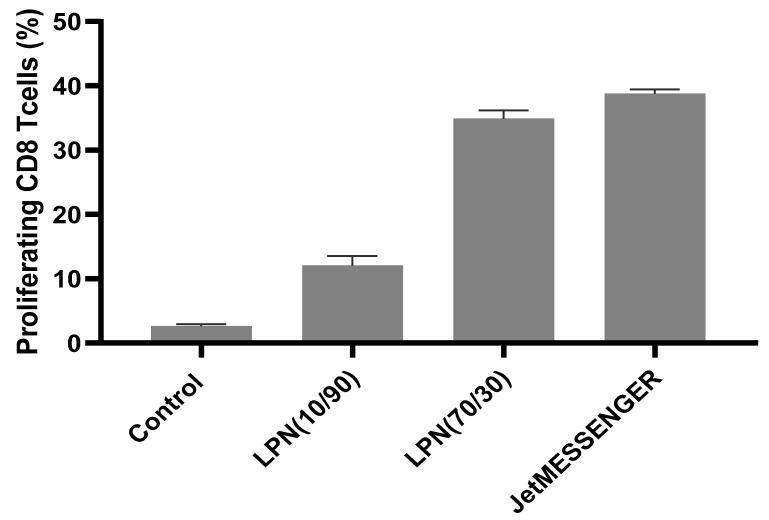
Proliferation of OVA-specific murine CD-8+ T-cells after stimulation by primary DCs. Successful transfection of DCs with mRNA loaded LPNs promoted effective activation and proliferation of antigen-specific T-cells. mRNA encodes for the model antigen ovalbumin that fits the specificity of the added T-cells. LPN(70/30) with DOPE content of 70% in the lipid layer again outperforms LPN(10/90) with higher DOTMA content.

In the next step, we further challenged the delivery system using bone marrow derived dendritic cells. We selected the LPN(70/30) and as comparison again LPN(10/90) and loaded both with mRNA encoding for the model antigen ovalbumin (OVA). In this assay the endpoint was not to look at the expression of a fluorescent reporter protein but rather at the activation/proliferation of antigen specific T-cells. Indeed, after successful transfection of the BMDC followed by translation, processing and presentation of the antigen in the context of the major histocompatibility complex I (MHC-I), the BMDC should be able to induce the activation and proliferation of OVA-specific CD8+ T-cells. This proliferation was assessed by flow cytometry. Both, the JetMESSENGER control and the LPNs led to proliferation of CD8-T-cells that was higher than the one elicited by the medium control (Figure 9). LPN(70/30) stimulated almost three times more cells than the LPN(10/90) confirming the good performance of these LPNs in the experiments with the DC2,4 cells. We did not find any proliferation of OVA-specific CD4-T-cells.

## 4. Discussion

Most of the presented experiments showed an effect of the lipid composition with a linear trend beginning with LPN(0/100) and peaking around LPN(70/30) except for mRNA binding and protection. In most cases, LPN(90/10) and LPN(100/0) ended this trend behaving differently.

We observed an increase in the zeta potential of the plain LPNs when increasing the DOPE content reaching the highest value at LPN(70/30), for which we do not have a clear explanation. The lipids may be tightly packed so that the negative phosphate of the DOPE may be covered completely and thus not influence the zeta potential measurement anymore, or the structural arrangement of the lipids on the particle surface is altered with changing ratio.

Looking at the mRNA binding efficacy, we found a continuous trend that the more DOPE the LPNs contained, the more mRNA was accessible to dyes after the loading process. For the last nanocarrier in the row, the LPN(100/0), the calculated BE was only 20%. From the work of Oude Blenke et al. we know that quantification results can vary considerably and are thus not always comparable [11]. In our case, the low binding efficacy is explainable as the zetapotential measurement before loading of this DOPE-only LPN revealed a slightly negative value (−12 mV). The primary amine group has a positive charge for pH values below 8 compensating the negative phosphate [12] as none of the used buffers or biological fluids exceeded a pH of 7.4. Due to the zwitterionic molecular structure of the phospholipid DOPE, we can conclude that the LPN(100/0) has no surface charge limiting electrostatic interaction with the mRNA. This lack of positively charged binding partners on the nanocarriers’ surface explains all our observations like the low binding efficacy, the low protection and finally the absence of protein expressing cells in transfection experiments. We can therefore conclude that a least 10% of DOTMA (mol% in lipid layer) is necessary for the mRNA complexation because DOPE does not contribute as much to the mRNA binding. Kranz et al. observed a similar phenomenon with better performing mRNA loaded liposomes (lipoplexes) if the overall charge is negative until the trend ended with higher amounts of unbound mRNA [13].

Gel electrophoresis confirmed the hypothesis that a minimum content of DOTMA is needed. Up to a DOPE content of 40% (LPNs (0/100–40/60)) ethidium bromide did not give any detectable signal in the gel. The addition of heparin released at least part of the bound mRNA showing a clear signal. Thus, we can conclude that the mRNA was there before but was bound too tightly to the nanoparticles’ surface for the dye to intercalate. The control sample with naked mRNA showed a fainter signal in the gel after heparin exposure even though loaded mRNA amounts were the same for heparin treated and untreated samples. We assume this reduced signal intensity is caused by an unintended degradation of the mRNA during the additional 30 min incubation time of heparin by ubiquitous nucleases. Our RNase exposure experiment confirmed the high sensitivity and fast degradation process of the naked mRNA. Most LPNs showed a mRNA band after the release that was brighter than the one of the control sample giving hints for a certain degree of protection by LPN complexation that was also confirmed in the RNase exposure study.

The phenomenon of lacking mRNA signal before heparin treatment for LPNs with high DOTMA amount was also observed for the fluorescence dye RiboGreen^®^ that revealed the same trend. As first nanocarrier in the row, LPN(50/50) did have some signal in the pocket but without unbound mRNA in the gel. The pocket signal was stronger for LPN(70/30) and can be explained by a fraction of less tight bound mRNA. With only 30% of DOTMA in the lipid layer, there are not enough binding partners for all mRNA molecules resulting in nucleic acids that are loosely attached to the particles but also accessible to intercalating dyes. This way of complexation seems to be enough to protect the mRNA from degradation of the RNases as shown in the RNase challenge experiment. The completely unbound mRNA will be degraded, but the attached molecules at the surface are safe probably due to steric hindrance. Therefore, we recommend performing such a challenge even though our setup has some limitations. Firstly, the used RNase A concentration of 0.0013 K/µg mRNA or 0.065 U/µg mRNA is low enough to enable the observation of the degradation process but comparable to other published protocols [14]. It has long been known that RNases are found in most biological fluids such as blood plasma or saliva but the quantification of their activity remains difficult [15,16,17]. Zhang et al. found high activities against naked mRNA in human blood serum and estimated a half lifetime of 1–2 min, indicating higher concentrations than used in our assay.

Secondly, for all tested LPNs, heparin addition resulted in a signal in the pocket and in the gel but we were not able to detach all the mRNA. This applies also for the RNase protection assay with RiboGreen^®^, when heparin increased the signal only for a part of the LPNs. Thus, a quantitative readout is not possible but rather a comparison of the nanoparticles. Alternatively, E. G. Bligh et al. established a protocol to directly measure the encapsulated nucleic acid amount by destroying lipid-based nanoparticles and extracting the mRNA [18]. Due to the high stability of the polymer core of the presented particles, it was not possible to adapt that protocol.

In the cell-based experiments, all LPNs were tolerated well by the dendritic cells in both media conditions (±supplementation with 10% FCS, HEPES-buffer, non-essential amino acids and β-mercaptoethanol). We saw the expected trend that nanocarriers with a higher DOTMA content have a certain cytotoxic potential that is reduced as the DOTMA amount decreases. We found more dead cells in the non-supplemented medium, as suboptimal nutrition made the cells more sensitive. This is in alignment with the findings of Uchida et al. who showed a reduced toxicity for different cell lines if DNA transfection is performed in serum containing media [19]. In the supplemented medium, all LPNs were comparably well tolerated without any differences because of the lipid composition. The incubation in the regular growth medium for 24 h was only possible due to the high colloidal stability of the LPNs that is connected with the PLGA core. The polymeric part of the nanoparticles enables furthermore a storage in the fridge for several weeks while maintaining the hydrodynamic and transfection properties.

When LPN(0/100) get in contact with protein containing media, the nanoparticles immediately agglomerate and precipitate. As soon as DOPE is part of the lipid layer, the nanocarriers maintain their monodispersity also in more complex media and are thus suitable for incubation times in such dispersants. For cell culture experiments, the precipitation might even be beneficial for the outcome as nanocarriers microprecipitate on the cells resulting in a higher concentration at the cells surfaces. DC2.4 cells are professional antigen presenting cells (APCs) and are therefore able to take up even such large agglomerates, which probably led to protein expression in our assays. This unique property of APC may result in an overestimation of the in vitro transfection efficiency with a high risk of failure if these LPNs (LPN(0/100)) are later applied in vivo.

The addition of DOPE did not only lead to colloidal stability in more complex media but did also increase the uptake, which is in concordance with the work of Farhood et al. [8]. We saw that effect especially for LPNs with lower DOPE content (0/100–30/70), where the phospholipid increased the uptake from 50% (LPN(0/100)) to 87% (LPN(30/70) in non-supplemented medium. The DOPE-only LPNs (LPN(100/0)) had again an uptake of around 58% indicating that the negative surface charge might hinder uptake.

Looking at the transfection results revealed that the uptake is not the crucial step for the presented LPNs. Even though the uptake is above 85% for LPNs (30/70–90/10) in supplemented medium, the transfection efficacy is very different. The results obtained in the RNase protection assay, excluded the possibility that the cargo is degraded by nucleases in the fetal calf serum. Thus, the missing piece might be the nanocarriers’ ability of escaping the endosomes that we did not quantify. Furthermore, medium components such as proteins and salts, might bind to the surface and change the nanocarriers’ properties like size or charge. Wang et al. observed that serum proteins form a corona on positively charged polystyrene particles changing particle properties towards enhanced lysosomal degradation and thus protection of the cells [20]. These proteins could also cover positively charged LPNs (0/40–40/60) and explain their low transfection rates in supplemented medium. As APCs the used dendritic cell will still be able to take them up, but the LPNs will not end up in endosomes or at least will not escape them in sufficient amounts. Most of the mRNA cargo will therefore be degraded resulting in less transfected cells. It was previously shown that the effect of serum proteins depends on the components of the carrier ranging from a reduction to an increased transfection efficacy [21] and that it should be evaluated.

The optimal combination of lipids for our lipid–polymer nanoparticles was the LPN(70/30) showing similarly good results in both medium conditions. In comparison with LPN(10/90) this nanocarrier performed much better also in the primary cell stimulation experiment. This is particularly relevant in terms of biological activity, since this assay measures the capacity of the transfected cells to not only express the transgene, but also to process and present the resulting protein to antigen-specific T-cells. Still the commercial control JetMESSENGER stimulated more CD8+ T-cells maybe because the produced amount of ovalbumin per cells and thus the activation of the transfected DC was higher. It needs to be tested if the measured stimulation differences between 35% and 39% (LPN(70/30) and JetMESSENGER, respectively) elicits a sufficient immune reaction and if these results are transferable to potential applications in vivo. Based on the improved stability in combination with good transfection efficacy and superior biocompatibility, LPNs might eventually outperform some commercial transfection reagents which have been optimized only for cell culture-based assays.

## 5. Conclusions

In this study, we could demonstrate that a DOPE content of between 50–80 mol% in a cationic lipid layer, here DOTMA, surrounding the PLGA core adds benefits for uptake and mRNA transfection efficacy of the lipid–polymer nanoparticles (LPN). Furthermore, our observations confirmed that it is worth to test not only in “simple” media with a low serum content, but to challenge the nanocarriers early and select later after testing in more complex surroundings. Based on a comprehensive portfolio of in vitro experiments, we found that the composition of lipids surrounding the PLGA core may be critical for transfecting dendritic cells in a biorelevant medium, with an optimum at 70% DOPE and 30% DOTMA.

## Data Availability

Data of the study is included in the paper or supplement. Raw data is available from the authors upon reasonable request.

## Supplementary Materials

The following supporting information can be downloaded at: https://www.mdpi.com/article/10.3390/pharmaceutics14122675/s1. Table S1: Colloidal properties of plain fluorescently labeled LPNs measured by dynamic light scattering. Figure S1: Size distributions of five different batches of LPN(70/30) showing the reproducibility of the nanocarrier production. Each graph represents one out of three measurements that were taken per batch with dynamic light scattering. Figure S2: Viability assay of plain LPNs dispersed in HBSS in increasing concentrations analyzed with DAPI staining and flow cytometry. 4 h incubation of LPNs in HBSS with DC2.4 cells. HBSS without any nanoparticles was used as live control. All tested LPNs were tolerated well by the DCs indicated by measured cell viability values above 75%. Figure S3: Comparison of cytotoxic effects of plain and mRNA loaded LPNs after 24 h incubation in RPMI medium without supplements. DAPI staining and flow cytometry were used for analysis. We used medium without nanoparticles as live control, 5% ethanol in HBSS (*v*/*v*) as dead control. Independent of the loading status, all LPNs were tolerated well by the cells. mRNA loading increased the viability even further for some LPNs. Figure S4: Gating strategy for the DC2.4 live cell population. mCherry-mRNA signal was measured in PE-Texas Red channel, DAPI signal in Pacific Blue channel and uptake of FITC-labeled LPNs in FITC channel. Figure S5: LPN uptake quantified by measured fluorescence intensities of FITC-labeled LPNs into DC2.4 cells following 24 h incubation. LPNs with high (>90%) DOTMA or DOPE content show lowest uptake. Figure S6: Transfection efficacy by measured fluorescence intensities of mCherry signal in DC2.4 after 24 h of incubation with the LPNs. LPN(70/30) shows highest protein levels in supplemented medium that is comparable to the performance in non-supplemented medium. Figure S7. Stability of selected mRNA loaded DiD-labeled LPNs in demineralized water and supplemented medium (supplemented with 10% FCS, HEPES-buffer, non-essential amino acids and β-mercaptoethanol). A: LPN(10/90), B: LPN(70/30). Nanoparticle Tracking Analysis showed no hints for instabilities in none of the media.

## References

[1] Abtin A., Eckhart L., Mildner M., Ghannadan M., Harder J., Schröder J.-M., Tschachler E. (2009). Degradation by stratum corneum proteases prevents endogenous RNase inhibitor from blocking antimicrobial activities of RNase 5 and RNase 7. J. Investig. Dermatol..

[2] Rademacher F., Dreyer S., Kopfnagel V., Gläser R., Werfel T., Harder J. (2019). The Antimicrobial and Immunomodulatory Function of RNase 7 in Skin. Front. Immunol..

[3] Yen A., Cheng Y., Sylvestre M., Gustafson H.H., Puri S., Pun S.H. (2018). Serum Nuclease Susceptibility of mRNA Cargo in Condensed Polyplexes. Mol. Pharm..

[4] Dirisala A., Uchida S., Tockary T.A., Yoshinaga N., Li J., Osawa S., Gorantla L., Fukushima S., Osada K., Kataoka K. (2019). Precise tuning of disulphide crosslinking in mRNA polyplex micelles for optimising extracellular and intracellular nuclease tolerability. J. Drug Target..

[5] Su X., Fricke J., Kavanagh D.G., Irvine D.J. (2011). In vitro and in vivo mRNA delivery using lipid-enveloped pH-responsive polymer nanoparticles. Mol. Pharm..

[6] Siewert C.D., Haas H., Cornet V., Nogueira S.S., Nawroth T., Uebbing L., Ziller A., Al-Gousous J., Radulescu A., Schroer M.A. (2020). Hybrid Biopolymer and Lipid Nanoparticles with Improved Transfection Efficacy for mRNA. Cells.

[7] Terada T., Kulkarni J.A., Huynh A., Tam Y.Y.C., Cullis P. (2021). Protective Effect of Edaravone against Cationic Lipid-Mediated Oxidative Stress and Apoptosis. Biol. Pharm. Bull..

[8] Farhood H., Serbina N., Huang L. (1995). The role of dioleoyl phosphatidylethanolamine in cationic liposome mediated gene transfer. Biochim. Biophys. Acta (BBA)-Biomembr..

[9] Yasar H., Biehl A., de Rossi C., Koch M., Murgia X., Loretz B., Lehr C.-M. (2018). Kinetics of mRNA delivery and protein translation in dendritic cells using lipid-coated PLGA nanoparticles. J. Nanobiotechnol..

[10] Weiss B., Schaefer U.F., Zapp J., Lamprecht A., Stallmach A., Lehr C.-M. (2006). Nanoparticles made of fluorescence-labelled Poly(L-lactide-co-glycolide): Preparation, stability, and biocompatibility. J. Nanosci. Nanotechnol..

[11] Oude Blenke E., Evers M.J.W., Baumann V., Winkler J., Storm G., Mastrobattista E. (2018). Critical evaluation of quantification methods for oligonucleotides formulated in lipid nanoparticles. Int. J. Pharm..

[12] Litzinger D.C., Huang L. (1992). Phosphatodylethanolamine liposomes: Drug delivery, gene transfer and immunodiagnostic applications. Biochim. Biophys. Acta (BBA)-Rev. Biomembr..

[13] Kranz L.M., Diken M., Haas H., Kreiter S., Loquai C., Reuter K.C., Meng M., Fritz D., Vascotto F., Hefesha H. (2016). Systemic RNA delivery to dendritic cells exploits antiviral defence for cancer immunotherapy. Nature.

[14] Zhang H., Rombouts K., Raes L., Xiong R., de Smedt S.C., Braeckmans K., Remaut K. (2020). Fluorescence-Based Quantification of Messenger RNA and Plasmid DNA Decay Kinetics in Extracellular Biological Fluids and Cell Extracts. Adv. Biosyst..

[15] Weickmann J.L., Glitz D.G. (1982). Human ribonucleases. Quantitation of pancreatic-like enzymes in serum, urine, and organ preparations. J. Biol. Chem..

[16] Potenza N., Salvatore V., Migliozzi A., Martone V., Nobile V., Russo A. (2006). Hybridase activity of human ribonuclease-1 revealed by a real-time fluorometric assay. Nucleic Acids Res..

[17] Lu L., Li J., Moussaoui M., Boix E. (2018). Immune Modulation by Human Secreted RNases at the Extracellular Space. Front. Immunol..

[18] Bligh E.G., Dyer W.J. (1959). A rapid method of total lipid extraction and purification. Can. J. Biochem. Physiol..

[19] Uchida E., Mizuguchi H., Ishii-Watabe A., Hayakawa T. (2002). Comparison of the efficiency and safety of non-viral vector-mediated gene transfer into a wide range of human cells. Biol. Pharm. Bull..

[20] Wang F., Yu L., Monopoli M.P., Sandin P., Mahon E., Salvati A., Dawson K.A. (2013). The biomolecular corona is retained during nanoparticle uptake and protects the cells from the damage induced by cationic nanoparticles until degraded in the lysosomes. Nanomedicine.

[21] Reiser A., Woschée D., Mehrotra N., Krzysztoń R., Strey H.H., Rädler J.O. (2019). Correlation of mRNA delivery timing and protein expression in lipid-based transfection. Integr. Biol. (Camb).

