# Supplementary material for: Lipid–Polymer Hybrid Nanoparticles for mRNA Delivery to Dendritic Cells: Impact of Lipid Composition on Performance in Different Media
Source: Pharmaceutics. 2022 Dec 1;14(12):2675. doi: 10.3390/pharmaceutics14122675 (PMC9782540; doi:10.3390/pharmaceutics14122675)
Supplement: Supplementary file 1 [file pharmaceutics-14-02675-s001.zip › pharmaceutics-2041310-supplementary.pdf]

pharmaceutics

Lipid-Polymer Hybrid Nanoparticles for mRNA Delivery to Dendritic Cells: Impact of Lipid Composition on Performance in Different Media

Lena Kliesch [1,2], Simon Delandre [3], Aljoscha Gabelmann [1,2], Marcus Koch [4], Kai Schulze [3], Carlos A. Guzmán [3], Brigitta Loretz [1,2]* and Claus-Michael Lehr [1,2]

[1] Helmholtz-Institute for Pharmaceutical Research Saarland (HIPS), Helmholtz Centre for Infection Research, Germany, Campus E8.1, 66123 Saarbrücken, Germany
[2] Department of Pharmacy, Saarland University, 66123 Saarbrücken, Germany
[3] Department of Vaccinology and Applied Microbiology, Helmholtz Centre for Infection Research, Inhoffenstraße 7, 38124 Braunschweig, Germany
[4] INM - Leibniz-Institut für Neue Materialien, Campus D2 2, 66123 Saarbrücken, Germany
* Correspondence: brigitta.loretz@helmholtz-hips.de; Tel.: +49-681-98806-1030

Supplementary Materials

Table S1. Colloidal properties of plain fluorescently labeled LPNs measured by dynamic light scattering

LPN	Marker	Size [nm]	PDI	Zetapotential [mV]	Batches tested
LPN(0/100)	-	194.43 ± 11.02	0.09 ± 0.02	21.67 ± 1.11	5
	FITC	200.30 ± 9.78	0.10 ± 0.03	24.79 ± 3.73	3
LPN(10/90)	-	200.49 ± 8.49	0.10 ± 0.02	22.49 ± 3.38	5
	DiD	198.60 ± 0.96	0.12 ± 0.03	29.7 ± 2.50	1
	FITC	197.09 ± 9.50	0.09 ± 0.02	25.87 ± 3.85	4
LPN(20/80)	-	208.73 ± 8.86	0.09 ± 0.02	26.33 ± 2.17	5
	FITC	225.06 ± 20.31	0.13 ± 0.07	28.73 ± 2.34	3
LPN(30/70)	-	215.69 ± 16.30	0.10 ± 0.02	29.71 ± 4.14	5
	FITC	209.87 ± 13.13	0.10 ± 0.02	28.66 ± 4.21	3
LPN(40/60)	-	219.84 ± 17.59	0.11 ± 0.03	30.28 ± 2.07	5
	FITC	237.69 ± 3.72	0.13 ± 0.06	32.41 ± 4.67	3
LPN(50/50)	-	229.15 ± 20.51	0.12 ± 0.03	32.90 ± 2.68	5
	FITC	243.22 ± 9.74	0.13 ± 0.02	34.74 ± 1.39	3
LPN(70/30)	-	219.77 ± 11.35	0.14 ± 0.02	38.26 ± 1.72	5
	DiD	218.85 ± 10.08	0.12 ± 0.04	28.56 ± 8.13	4
	FITC	243.93 ± 13.00	0.13 ± 0.03	34.00 ± 2.20	5
LPN(100/0)	-	205.86 ± 12.64	0.12 ± 0.02	-11.75 ± 0.69	5
	FITC	237.56 ± 26.28	0.14 ± 0.07	-9.77 ± 5.13	3

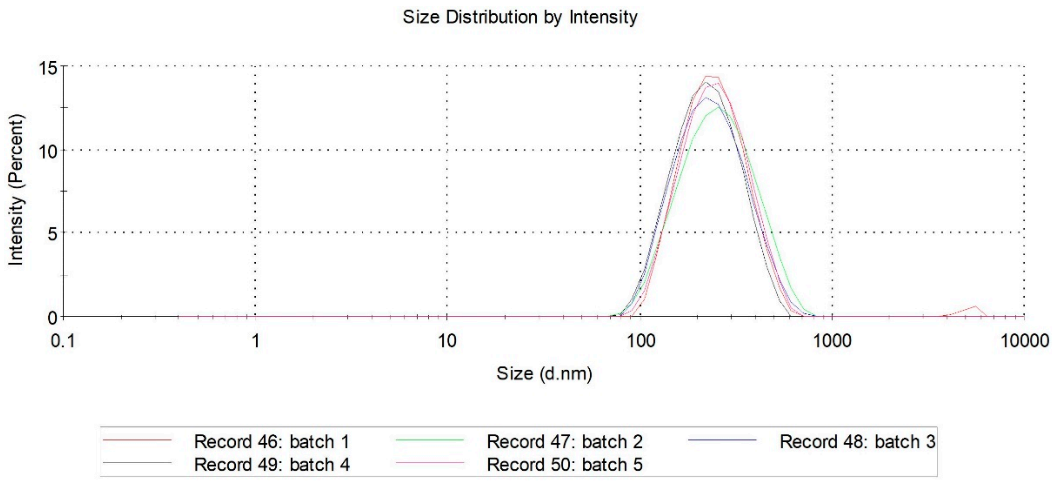

Figure S1. Size distributions of five different batches of LPN(70/30) showing the reproducibility of the nanocarrier production. Each graph represents one out of three measurements that were taken per batch with dynamic light scattering.

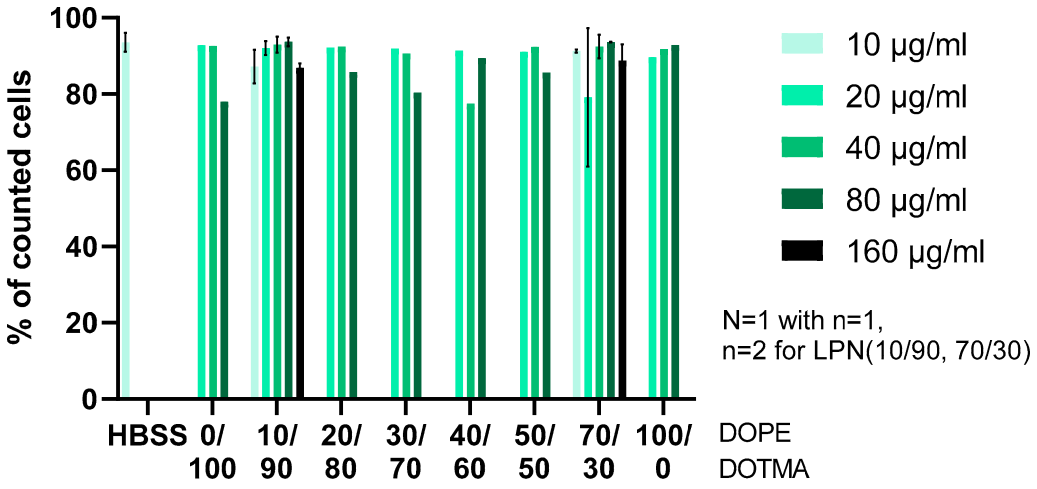

Figure S2. Viability assay of plain LPNs dispersed in HBSS in increasing concentrations analyzed with DAPI staining and flow cytometry. 4 h incubation of LPNs in HBSS with DC2.4 cells. HBSS without any nanoparticles was used as live control. All tested LPNs were tolerated well by the DCs indicated by measured cell viability values above 75%.

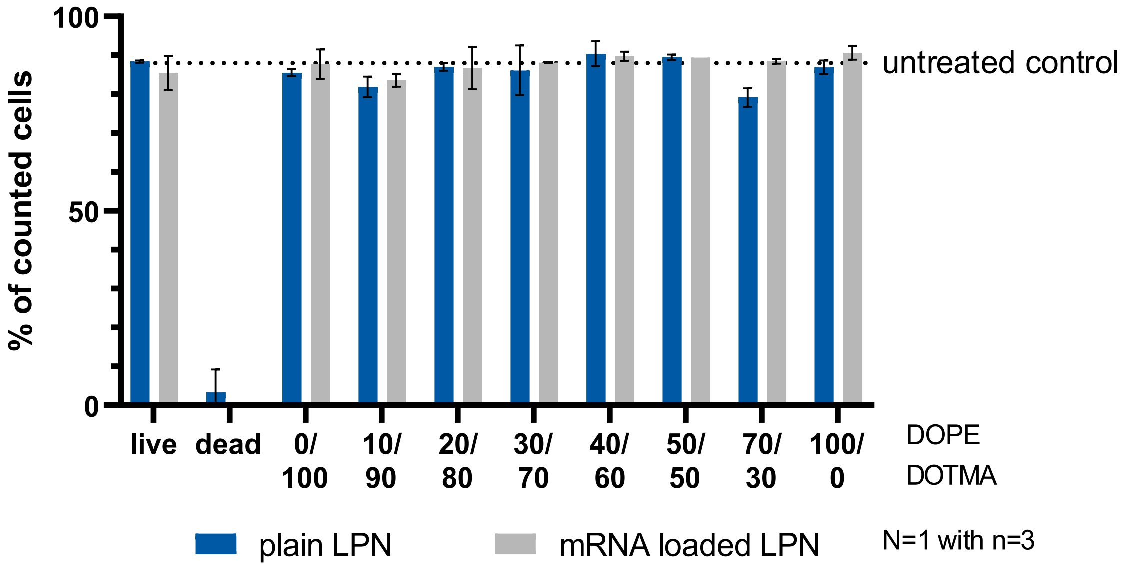

Figure S3. Comparison of cytotoxic effects of plain and mRNA loaded LPNs after 24 h incubation in RPMI medium without supplements. DAPI staining and flow cytometry were used for analysis. We used medium without nanoparticles as live control, 5% ethanol in HBSS (v/v) as dead control. Independent of the loading status, all LPNs were tolerated well by the cells. mRNA loading increased the viability even further for some LPNs.

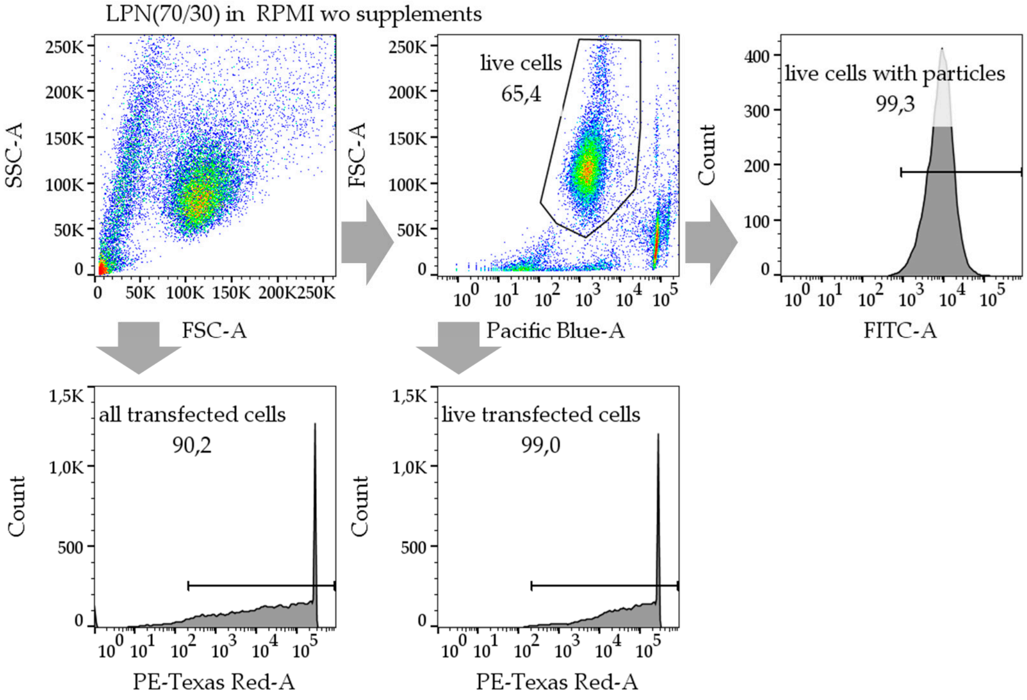

Figure S4. Gating strategy for the DC2.4 live cell population. mCherry-mRNA signal was measured in PE-Texas Red channel, DAPI signal in Pacific Blue channel and uptake of FITC-labeled LPNs in FITC channel.

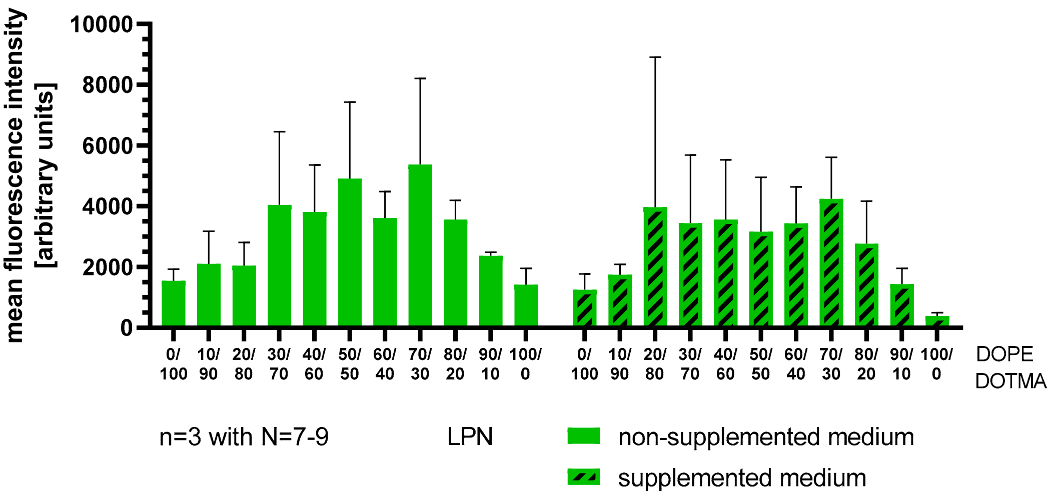

Figure S5. LPN uptake quantified by measured fluorescence intensities of FITC-labeled LPNs into DC2.4 cells following 24 h incubation. LPNs with high (>90%) DOTMA or DOPE content show lowest uptake.

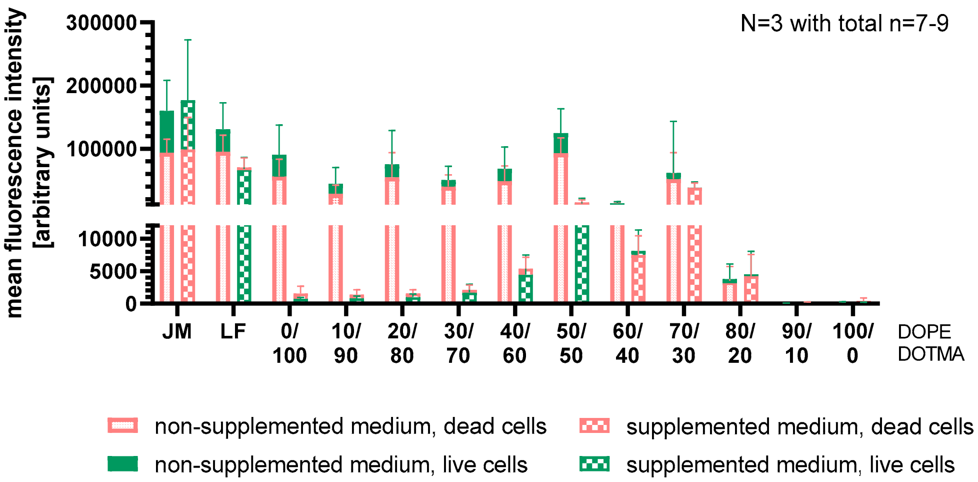

Figure S6. Transfection efficacy by measured fluorescence intensities of mCherry signal in DC2.4 after 24 h of incubation with the LPNs. LPN(70/30) shows highest protein levels in supplemented medium that is comparable to the performance in non-supplemented medium.

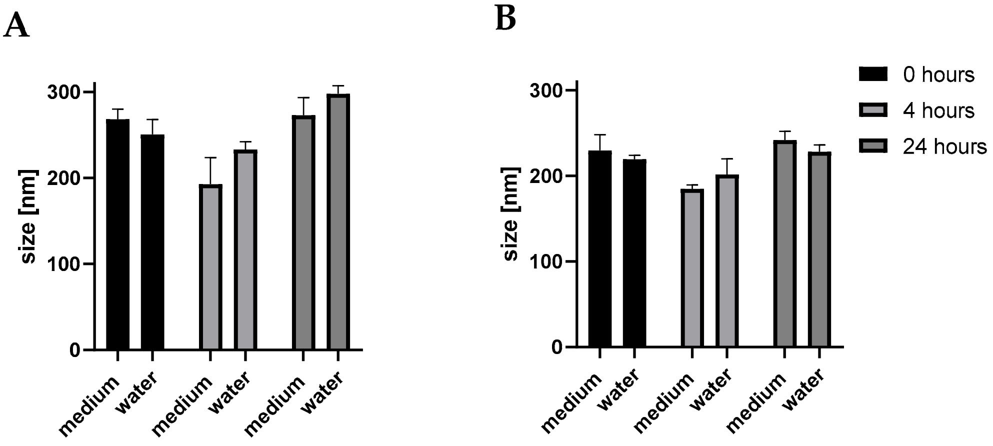

Figure S7. Stability of selected mRNA loaded DiD-labeled LPNs in demineralized water and supplemented medium (supplemented with 10% FCS, HEPES-buffer, non-essential amino acids and β-mercaptoethanol). A: LPN(10/90), B: LPN(70/30). Nanoparticle Tracking Analysis showed no hints for instabilities in none of the media.
